# Editorial: Plant-water relations for sustainable agriculture

**DOI:** 10.3389/fpls.2022.979804

**Published:** 2022-07-28

**Authors:** Thorsten Knipfer, Italo F. Cuneo

**Affiliations:** ^1^Faculty of Land and Food Systems, The University of British Columbia, Vancouver, BC, Canada; ^2^Facuty of Agriculture and Food Science, Pontificia Universidad Católica de Valparaíso, Valparaíso, Chile

**Keywords:** plant hydraulics, water relations, irrigation, water use efficiency, drought

Ongoing climate change causes unprecedented challenges for agriculture. The modern farmer requires advanced knowledge about plant stress physiology to avoid losses in crop production and quality. This Research Topic on “Plant water relations for Sustainable Agriculture” provides advanced understanding of the mechanisms and responses of crops to water stress by drought and waterlogging.

Crop performance depends on a successful coordination of physiological, anatomical, molecular and morphological processes at root, stem, and leaf level. In this issue, Gervais et al. conducted a large screening study of potato drought stress performance. Authors found differences in tuber yield between drought tolerant and susceptible genotypes and an increase in water use efficiency (WUE) by 2–3-fold in more drought tolerant genotypes. Similarly, Martínez et al. identified differences in physiological performance and productivity in response to soil water availability (i.e., full irrigation and rainfed conditions) among several potato varieties. They found that water stress negatively affects tuber size distribution, reducing overall production by 50–60%. In an effort to improve the selection of drought tolerant rice genotypes, Mahreen et al. combined morpho-physiological and biochemical approaches with infrared thermal imaging technology. Using reliable, fast, and non-destructive technology may accelerate the selection of drought tolerant genotypes in breeding programs. Zheng et al. shows that improving root morphological traits and alleviating root senescence during mid-grain filling are important characteristics that could be used in wheat breeding programs targeting high yield and water use efficiency. For different maize hybrids, Alam et al. reported on the effect of different levels of drought stress on kernel water relations and filling in a tropical environment. On a molecular level, the study of Liang et al. points toward a better understanding of metabolic pathways for improving water management. The authors present a detailed metabolomic depiction of the importance of soil arbuscular mycorrhizal fungi that enhances drought tolerance in trifoliate orange. Similarly, and also in trifoliate orange, Cheng et al. studied the effect of arbuscular mycorrhizal fungi in regulating H+-ATPase activity and gene expression. They showed that inoculated plants exhibit greater photosynthetic rate, stomatal conductance, and better root characteristics than non-mycorrhizal plants. In contrast, Liu D. et al. reported on problems of water stress by flooding in peanut. The authors used proteomics analysis, and found different metabolic mechanisms that affect the accumulation of toxic substances and enhance anaerobic respiration activity of enzymes. Together, this knowledge facilitates the selection of crop genotypes with improved drought tolerance and the development of sustainable irrigation practices.

Irrigation strategies should be informed by key aspects of plant hydraulics. In this issue, Luo et al. presents a leaf hydraulics model that captures spatiotemporal changes of water potential and flow rates in monocotyledonous and dicotyledonous leaves. The authors point to a substantial contribution of leaf capacitance and resistances that should be acknowledged when interpreting leaf hydraulics in the context of water management on a field scale. For horticultural trees, Sheridan and Nackley used traditional plant hydraulic methods to investigate their desiccation during prolonged cold storage. Their results provide key information to nurseries for improving plant management.

Crop water requirements change daily and annually based on environmental conditions. The pressure chamber provides a tool for measuring plant water status in a cost-effective and robust way. For olive trees, Shackel et al. presents a large data set on stem water potentials and vapor pressure deficit collected over multiple years. The authors established a reference baseline for olives that will allow growers to utilize plant-based irrigation management at olive orchard level. On a related topic, Zhang et al. shows how vapor pressure deficit affects photosynthetic carbon dioxide uptake in a process mediated by foliar abscisic acid content in tomato. This study provides basic information related to the complex water relations at leaf level and how they affect whole plant water relations. Measurements of soil moisture can serve as an indirect indicator of plant water status. Niu et al. used a specific water system to maintain a stable soil moisture level, improving water use efficiency in maize subjected to short-term soil water stress.

For crop production, the meaning of “drought” can be many-fold, but our goal must be to identify ways to maintain production under water-limiting conditions while protecting water as one of our most important natural resources. On this, management becomes crucial, for example, Liu R. et al. presents data that shows that alternate partial root-zone drip nitrogen fertigation improves water use efficiency while reducing the loss of residual nitrate in the soil profile. Also, data presented in this issue shows that specific soil management, such as subsoiling plus organic fertilization, improves water use efficiency by positively impacting soil structure (Yang et al.). The design of orchards with the focus placed in water savings requires key physiological information. Selecting grapevine drought tolerant rootstocks is a complex decision for viticulturists. Villalobos-Soublett et al. present data from naturalized grapevine rootstocks from arid regions of Chile that allows to adapt vineyards to regions with low water availability while maintaining adequate physiological performance for crop production. Stress management at field level requires understanding of key physiological signals of stress such as the balance between reactive oxygen species (ROS) and the antioxidative system (AOS) in plants. Rane et al. provides a thorough review of the role of ROS–AOS relation that ultimately affect photosynthetic efficiency and growth in dryland agricultural crop plants.

Under a fast-changing environment, it is clear that the modern farmer is challenged to make appropriate decisions. This issue highlights that an advanced understanding of crop performance can improve the decision-making process, at least in part. Also, this issue highlights how diverse and multi-layered research in the plant water relations community is to find best solutions to secure crop performance and yield under conditions of water stress. The future will provide extreme challenges for agriculture with water availability as the main driver.

## Author contributions

All authors listed have made a substantial, direct, and intellectual contribution to the work and approved it for publication.

## Funding

This work was supported by an operating grant from the Natural Sciences and Engineering Research Council of Canada (NSERC, GR019368 awarded to TK).

## Conflict of interest

The authors declare that the research was conducted in the absence of any commercial or financial relationships that could be construed as a potential conflict of interest.

## Publisher's note

All claims expressed in this article are solely those of the authors and do not necessarily represent those of their affiliated organizations, or those of the publisher, the editors and the reviewers. Any product that may be evaluated in this article, or claim that may be made by its manufacturer, is not guaranteed or endorsed by the publisher.

